# Cholera outbreak in some communities in North-East Nigeria, 2019: an unmatched case–control study

**DOI:** 10.1186/s12889-023-15332-4

**Published:** 2023-03-07

**Authors:** Idowu O. Fagbamila, Muhammad A. Abdulkarim, Mabel K. Aworh, Belinda Uba, Muhammad S. Balogun, Patrick Nguku, Ajibji Y. Gandi, Ibrahim Abdullahi, Emmanuel C. Okolocha, Jacob K. P. Kwaga, Ndadilnasiya E. Waziri

**Affiliations:** 1 Nigeria Field Epidemiology and Laboratory Training Programme, Abuja, Nigeria; 2 African Field Epidemiology Network, Abuja, Nigeria; 3 Bauchi State Primary Health Care Development Agency, Bauchi, Nigeria; 4 Department of Veterinary Public Health, Faculty of Veterinary Medicine, Ahmadu Bello University, Zaria, Nigeria

**Keywords:** Cholera, Case–control, Risk factors, Outbreak, Nigeria

## Abstract

**Background:**

Cholera, a diarrheal disease caused by the bacterium *Vibrio cholerae*, transmitted through fecal contamination of water or food remains an ever-present risk in many countries, especially where water supply, sanitation, food safety, and hygiene are inadequate. A cholera outbreak was reported in Bauchi State, North-eastern Nigeria. We investigated the outbreak to determine the extent and assess risk factors associated with the outbreak.

**Methods:**

We conducted a descriptive analysis of suspected cholera cases to determine the fatality rate (CFR), attack rate (AR), and trends/patterns of the outbreak. We also conducted a 1:2 unmatched case–control study to assess risk factors amongst 110 confirmed cases and 220 uninfected individuals (controls). We defined a suspected case as any person > 5 years with acute watery diarrhea with/without vomiting; a confirmed case as any suspected case in which there was laboratory isolation of *Vibrio cholerae* O1 or O139 from the stool while control was any uninfected individual with close contact (same household) with a confirmed case. Children under 5 were not included in the case definition however, samples from this age group were collected where such symptoms had occurred and line-listed separately. Data were collected with an interviewer-administered questionnaire and analyzed using Epi-info and Microsoft excel for frequencies, proportions, bivariate and multivariate analysis at a 95% confidence interval.

**Results:**

A total of 9725 cases were line-listed with a CFR of 0.3% in the state. Dass LGA had the highest CFR (14.3%) while Bauchi LGA recorded the highest AR of 1,830 cases per 100,000 persons. Factors significantly associated with cholera infection were attending social gatherings (aOR = 2.04, 95% CI = 1.16–3.59) and drinking unsafe water (aOR = 1.74, 95% CI = 1.07–2.83).

**Conclusion:**

Attending social gatherings and drinking unsafe water were risk factors for cholera infection. Public health actions included chlorination of wells and distribution of water guard (1% chlorine solution) bottles to households and public education on cholera prevention. We recommend the provision of safe drinking water by the government as well as improved sanitary and hygienic conditions for citizens of the state.

**Supplementary Information:**

The online version contains supplementary material available at 10.1186/s12889-023-15332-4.

## Background

Cholera, an acute diarrhoeal disease of public health importance is caused by a bacillus *Vibrio cholerae*, either serogroup O1 or O139 affecting both children and adults [1, 2]. About 20% of those who are infected develop acute, watery diarrhea – 10–20% of these individuals develop severe watery diarrhea with vomiting. If these patients are not promptly and adequately treated, the loss of such large amounts of fluid and salts can lead to severe dehydration and death within hours [1, 3]. The case-fatality rate in untreated cases may reach 30–50% although treatment is straightforward (basically rehydration) and, when applied appropriately, should keep the case-fatality rate below 1% [2, 4]. Cholera is usually transmitted feco-orally through contaminated water or food and remains a risk in many countries. New outbreaks can occur sporadically in any part of the world where water supply, sanitation, food safety, and hygiene are inadequate [1–4]. Refugee settings and over-populated communities that are characterized by poor sanitation, unsafe drinking water, and increased person-to-person transmission account for the greatest risk of this disease. Mild and asymptomatic cholera accounts for 80% of cases with incubation periods ranging from two hours to five days. Preventing cholera from entering a community is impossible, however, early detection and confirmation followed by an appropriate response can prevent the spread within the community.

Cholera is an acute public health issue with the high potential to cause many deaths, spread quickly and eventually internationally, and seriously affect travel and trade. Therefore, response to the outbreak has to be well-coordinated, timely, and effective [3]. Planning and implementation of preparedness activities that will allow for effective management of future cholera outbreaks should be preceded by good response activities. A strong cholera preparedness plan and program is the best preparation for outbreaks in countries at risk of cholera, whether or not they have yet been affected, or countries in which seasonal recurrence of the disease may be expected [4]. Cholera is endemic in many countries including Nigeria and recent studies have reported that global warming creates a favorable environment for these gram-negative bacteria to thrive [5]. Although reports of the cholera epidemic in Nigeria have not been consistent, the disease is very dynamic and appears to be endemic in Northern Nigeria. The cholera report in Kano State, Northern Nigeria revealed that the frequency and distribution of recurrent cholera epidemics in the state from 2010 to 2019, were 1608, 778, 0, 1678, 7058, 1094, 226, 948, 2982 and 89 respectively [6, 7]. In Jos, North Central Nigeria, the literature showed that all isolated strains were *Vibrio cholerae* 01 Eltor of Inaba serotype [8]. The burden of cholera in Nigeria is more in Northern Nigeria, although, very little is known about the characteristics of the circulating strains. The outbreak of cholera associated with gastroenteritis and the attendant deaths in some regions in Nigeria brought to the forefront the vulnerability of poor communities and most especially children to the infection [9]. Cholera outbreaks were often attributed to rain which washed sewage into open wells and ponds, where people obtain water for drinking and household needs [5, 7]. Even though the epidemic was recorded in northern Nigeria, epidemiological evidence indicated that the entire country was at risk, with the postulation that the outbreak was due to hyper-virulent strains of the organism [10]. Nigeria as one of the cholera foci in the world is characterized by persistent outbreak situations [11]. On 28^th^ February 2019, the index case a 12-year-old boy from a Tsangaya (Almajiri) School in the Gwallaga community of Makama B ward of Bauchi LGA was presented to the Abubakar Tafawa- Balewa University Teaching Hospital (ATBUTH) Bauchi on account of sudden onset of watery stool and vomiting. He was successfully managed and discharged after two days of hospitalization. A few hours after his admission to ATBUTH, another set of two (2) students from Markazi Tsangaya School in the Kobi area of Hardo ward were hospitalized at the Bauchi State Specialist Hospital with only one survival and the first mortality recorded during the outbreak in the State. Sixteen out of the 20 wards in Bauchi LGA and eight other LGAs (Alkaleri, Bogoro, Dass, Darazo, Ganjuwa, Kirfi, Tafawa- Balewa, and Toro) recorded at least one case of the disease from 28^th^ February to 31^st^ August, 2019. An outbreak investigation team was immediately deployed on the 2^nd^ of March, 2019 by the Bauchi State Government with technical support from Field Epidemiology and Laboratory Training Programme (FELTP) with the objectives of verifying the diagnosis, describing the extent of the outbreak, identifying associated risk factors, and instituting appropriate control measures by strengthening case management.

## Materials and methods

### Study area


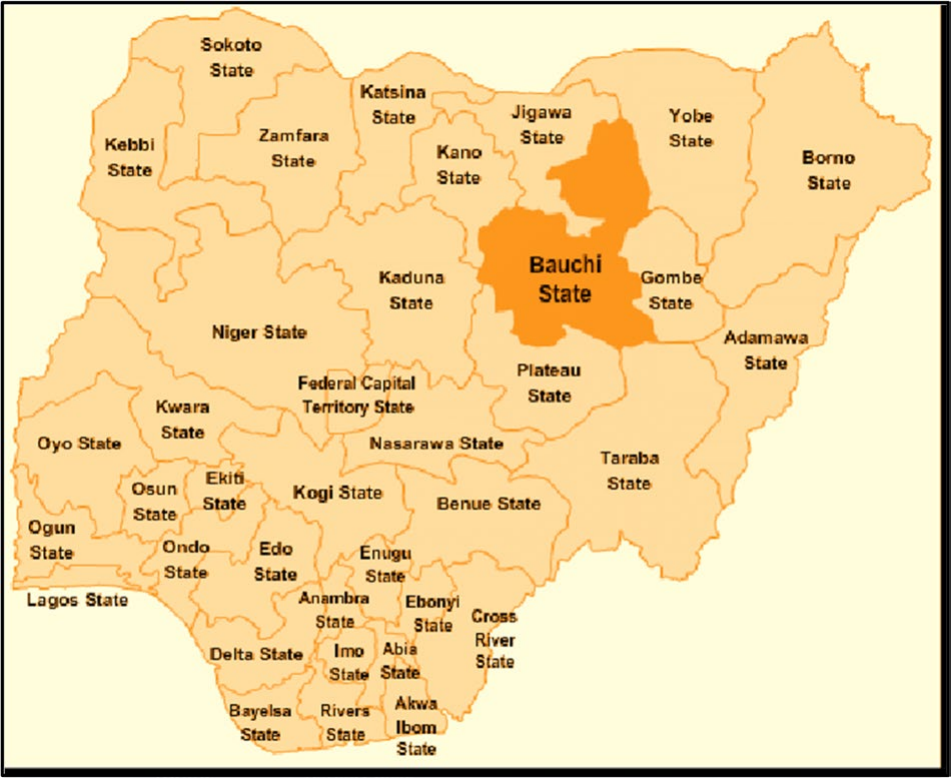


### Map of Nigeria showing the study area (Bauchi State)

The study was conducted in Bauchi State located in the North-East geopolitical zone of Nigeria with a surface area of 49,259 sq. km. Bauchi State is made up of twenty Local Government Areas (LGAs) with 323 Political Wards. Bauchi city serves as both Bauchi Local Government headquarters and the State Capital respectively. According to the 2006 census, Bauchi state has a population of 4,653,066.

### Notification of the outbreak

On 28 February 2019, few hours after the index case was hospitalized at the ATBU Teaching Hospital, the Epidemiology Unit of the Bauchi State Primary Health Care Development Agency (BASPHCDA) was notified by the Bauchi LGA Disease Surveillance and Notification Officer (DSNO). The State Rapid Response Team (RRT) was mobilized and dispatched to the affected communities/Tsangaya schools including the ATBU Teaching Hospital to investigate the outbreak. The RRT utilized several methods for the outbreak investigations including advocacy visits; active case searches at the health facilities and affected communities; verbal autopsy for retrospective deaths in the communities; review of case notes/patient registers at health facilities for retrospective cases; laboratory investigations and questionnaires administration.

### Study design

We conducted an unmatched case–control study to identify outbreak-associated risk factors. This study was carried out earlier (28^th^ Febraury-31^st^ March, 2019) in the outbreak, before identification and line listing of all the outbreak cases. For the case–control study, we identified 110 cholera cases from the line list of ongoing and recovering patients in the treatment centre. Contact tracing was conducted to identify and assess the geographical location of the infection sites as well as selecting controls and administering a semi open-ended questionnaire to them. For every case patient, two controls were identified.

#### Case definition

##### Suspected case

We defined a suspected case of cholera as *“any person or patient aged 5 years or more with acute watery diarrhea with or without vomiting living in Bauchi State from 28 February to 31 March 2019”*. To maintain specificity, therefore, children under 5 were not included in the case definition of cholera but samples from this age group were collected where such symptoms occurred and were separately line-listed.

##### Confirmed case

Confirmed case was defined *“as any suspected case in which Vibrio cholerae O1 or O139 has been isolated from stool at the laboratory”*.

#### Control definition

We defined a control as *“any person living in Bauchi State aged 5 years or more who is a friend or family member or neighbor of a case without any history of diarrhea from 28 February to 31 March 2019”*.

### Identification of cases and recruitment of controls

We selected all current cholera cases and those who had recovered from the line list. Using the case definitions, we conducted an active case search in the affected communities and reviewed health records at health facilities. All cases meeting the case definition were recruited. We extracted data on socio-demographic characteristics (sex, age, residence), date of onset of illness, date of presentation at the clinic, presenting signs and symptoms, history of treatment, and outcomes. Next, we generated hypotheses about possible exposure factors that were common to the cases. Controls were recruited from among family members (26%), friends (14.5%), and neighbors (59.5%) of the cases. Only persons who resided in the communities at least 10 days before the start of the outbreak and with no symptoms similar to the case definition during the stated period were considered eligible for selection as controls. Where more than two suitable controls were available for a case, only two were selected randomly.

### Case management

A cholera treatment centre at the ATBU Teaching Hospital Bauchi was set up to attend to all cholera cases in the state. This center was supported and managed by partners- *Medecins San Frontiers* (MSF). Referral and suspected patients in and around Bauchi LGA were brought in for management. The case identification procedures of all the health facilities in the affected wards were also reviewed.

### Data collection

#### Questionnaire survey

We used interviewer-administered questionnaires to collect the following information from cases and controls; socio-demographic characteristics, clinical information from cases, and possible exposure factors. The determination of risk factors for cholera infections involved the administration of questionnaires to assess the association between general hygiene vis-à-vis cholera infections amongst individuals in the communities where the outbreak occurred. Consent was sought before administering the questionnaire to cases and controls. Data collected on behavioral habits included house characteristics, source of water, fruits and vegetables, management of waste, hygienic practices such as cleaning and disinfection procedures, types of toilet facility, eating habits, number of individuals in a household, and date of onset of the disease. The questionnaire was designed and pretested on 20 individuals in and around Bauchi LGA and the questions were adjusted as necessary. However, data from the pretesting was not included in the final analysis.

#### Laboratory investigations

We used universal sterile bottles to collect stool samples for laboratory confirmation within 24 to 48 h of the onset of illness. Each appropriately labeled specimen was immediately transported to the nearest microbiology laboratory within one hour of collection where they were quickly processed for culture, gram staining, and antibiotic susceptibility testing. However, treatment of dehydrated patients was initiated immediately before the laboratory confirmation. We also collected and analyzed 9 water samples from wells and public water supply network in some affected communities.

#### Environmental assessment

Environmental assessment of the affected communities was conducted on open defecation, hygiene and sanitary practices, source of drinking water and sewage drainage system.

### Data analyses

All the data were collated and entered into an Excel 2007 (Microsoft) data base. Descriptive and statistical data analysis was carried out using Epi info version 7.0. Categorical variables were summarized as frequencies and proportions, while continuous variables (such as age), were expressed as mean and standard deviation. We calculated case fatality rates and developed a weekly epidemic curve to show the distribution of the cases by date (weeks) of onset of illness and date (weeks) of deaths. A bivariate analysis was conducted to identify risk factors associated with a cholera infection. Any variable significant at *p* < 0.05 was included in the multivariate logistic regression model in a forward and backward stepwise fashion. Variables were included or excluded from the model based on the adjusted Wald test statistics, and only variables with *p* < 0.05 were retained. For variables with multiple levels, one variable must be significantly different from the baseline (reference) for the variable to be retained. Possible confounders were controlled for by including these variables in the logistic regression model.

### Ethical considerations

We obtained written informed consent from study participants and caregivers of children before questionnaire administration as most of our respondents were uneducated. We assured the respondents of the confidentiality of information obtained. Permission was also obtained from the management of ATBU Teaching Hospital Bauchi and Bauchi State Primary Health Care Development Board before record review. The Research Ethics Committee of Abubakar Tafawa Balewa University (ATBU) waived ethical approval because of the exigencies of the outbreak response.

## Results

### Descriptive epidemiology of the cholera outbreak

The epidemiological investigation identified 9725 cases in ten LGA of Bauchi state and 6 cases from neighboring Plateau State. A total of 28 fatalities was recorded with a total case fatality rate (CFR) of 0.29%. Dass LGA had the highest CFR of 14.29%. Bauchi LGA, with a total population of 493, 810 based on the 2006 population census had the highest attack rate of 18,261 per 1,000,000 while Katagum and Alkaleri had the lowest attack rate of 4 per 1,000,000 (Table 1). Consequently, the Bauchi LGA had the highest percentage of all the cases recorded during the outbreak (92.7%) as well as the most hit in terms of the number of deaths.

**Table 1:** Case fatality rates and attack rates associated with 2019 Bauchi State Cholera outbreak

**LGA**	**Population (based on 2016 censors)**	**Number of cases**	**Death**	**Proportion of cases by LGA**	**Case fatality rate (%)**	**LGA attack rate/1,000,000**
Alkaleri	329, 424	1	0	0.01%	0.00	3.04
Bauchi	493, 810	9017	24	92.70%	0.27	18,260.06
Bogoro	84,215	10	0	0.10%	0.00	118.74
Darazo	251,597	2	0	0.02%	0.00	7.95
Dass	89,943	7	1	0.07%	14.29	77.83
Gajuwa	280,468	242	1	2.49%	0.41	862.84
Kirfi	147,618	1	0	0.01%	0.00	6.77
Tafawa Balewa	219,988	397	2	4.08%	0.5	1,804.56
Toro	350, 404	41	0	0.42%	0.00	117.01
Katagum	295,970	1	0	0.01%	0.00	3.38
Jos North (Plateau State)	429,300	6	0	0.06%	0.00	13.98
Total		9725	28	100.00%	0.29	

### Case control study

The study population comprised 330 participants (110 cases and 220 controls) with a response rate of 100%. Overall, the mean ages of the participants were 24.8 ± 12.9 years. Females were more 51.5% (*n* = 170); majority were students 44.9% (*n* = 148); had secondary education 42.1% (*n* = 139); and were not married 63.3% (*n* = 209). The mean age of cases and controls was 21.8 ± 12.5 years and 26.2 ± 12.9 years respectively. Cholera cases were slightly higher among females (60%) as compared to males. Infection in age group > 5–20 was higher (52.7%) when compared to age group 21 years and above (Table 2).

**Table 2:** Socio-demographic characteristics of cases and controls in Bauchi, 2019

**Variables**	**Cases *n (%)***	**Controls *n (%)***	**Total *n (%)***
**Total**	110 (33.3)	220 (66.7)	330 (100)
**Age (years)**			
> 5—20	58 (52.7)	88 (40)	146 (44.2)
21 and above	52 (47.3)	132 (60.0)	184 (55.8)
**Gender**			
Female	66 (60)	104 (47.3)	170 (51.5)
Male	44 (40)	116 (52.7)	160 (48.5)
**Marital Status**			
Single	79 (71.8)	130 (59.1)	209 (63.3)
Married	31 (28.2)	90 (40.9)	121 (36.7)
**Education**			
None	28 (25.4)	42 (19.1)	70 (21.2)
Primary	8 (7.3)	30 (13.6)	38 (11.5)
Secondary	51 (46.4)	88 (40)	139 (42.1)
Tertiary	23 (20.9)	60 (27.3)	83 (25.2)
**Occupation**			
None	29 (26.3)	63 (28.)	92 (27.9)
Student	63 (57.3)	85 (38.6)	148 (44.9)
Civil Servant	18 (16.4)	72 (32.8)	90 (27.2)

**Fig. 1:**
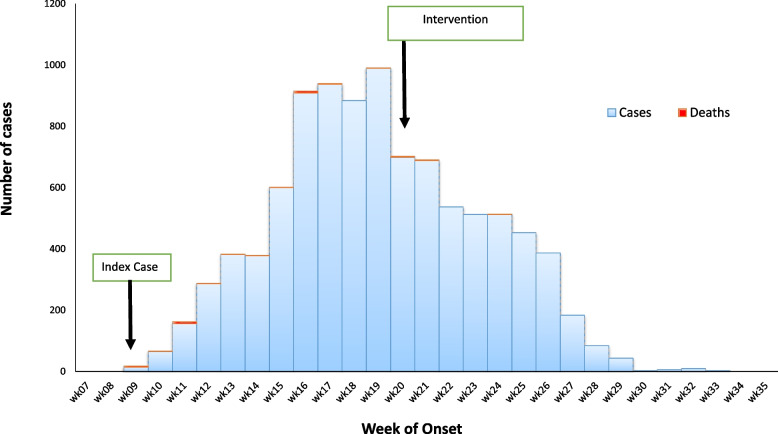
Epidemic curve of cholera cases by week of onset in Bauchi State from 11th February 2019 to 9th September 2019

The epidemic curve for the cholera outbreak is shown in Fig. 1. The index case was reported on 28 February 2019 (week 9 of 2019). A week after the index case was reported, the cholera treatment center was set up by the NGO to manage cases. There was a steady rise in the number of cases which eventually peaked by week 19. This coincided to the peak of dry season (heat period) characterized by scarcity of portable drinking water. However, the case fatality rate steadily declines during this same period. The peak of the outbreak was followed by a sharp decline at the beginning of second week of May, which coincided with the commencement of response activities by the Rapid Response Team in the state. This was followed by abrupt fall in the number of cases being reported. The outbreak ended at the end of 35^th^ week (14 days after the last case was admitted).

**Fig. 2:**
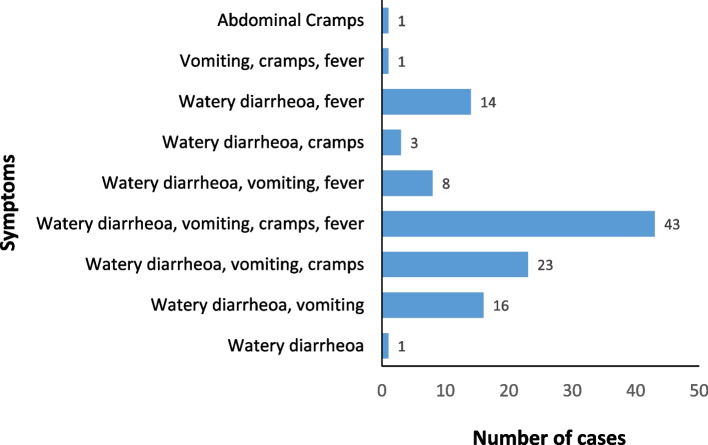
Symptoms of cholera cases in Bauchi, September 2019

The groups of symptoms frequently reported by the cases were watery diarrhoea, vomiting, abdominal cramps and fever (39.1%, *n* = 43); watery diarrhoea, vomiting and abdominal cramps (20.9%, *n* = 23) while one individual each reported watery diarrhoea and abdominal cramps as the only symptom they experienced during the outbreak (Fig. 2).

Out of 24 stool samples tested, 20 were confirmed positive for *Vibrio cholerae* using both rapid test kit and culture however the nine water samples obtained from wells and the public water supply network in the most affected communities were negative. Majority of individuals in both controls (87.7%), cases (94.5%) were more likely to dispose refuse in the backyard or bush or a combination of both than by burning (Table 3).

**Table 3:** Factors associated with Cholera outbreak at bivariate analysis, Bauchi, 2019

Variables	Cases	Control	OR	95% CI	*P*-value
**Gender**					
Female	66	104	**1.67**	**1.05–2.66**	**0.04**
Male	44	116			
**Occupation**					
None	29	63	1.84	0.93–3.63	0.11
Student	63	85	**2.96**	**1.61–5.46**	**0.001**
Civil servant/ business	18	72	Ref	Ref	
**Travelled 10 days prior to the disease?**					
Yes	38	52	**1.71**	**1.03–2.81**	**0.05**
No	72	168			
**Contact with diarrhea patient?**					
Don’t know	28	32	**1.86**	**1.03–3.36**	**0.05**
Yes	20	56	0.76	0.42–1.37	0.47
No	62	132	Ref	Ref	
**Attend social gathering?**					
Yes	46	55	**2.16**	**1.33–3.51**	**0.003**
No	64	165			
**Source of drinking water**					
Water vendor/pure water	27	56	1.52	0.71–3.23	0.37
Well	37	33	**3.52**	**1.64–7.56**	**0.002**
Combination of any of the above	32	87	1.16	0.56–2.39	0.83
Borehole/tap water	14	44	Ref	Ref	Ref
**Where do you buy fruits/vegetables?**					
Market	85	152	**2.41**	1**.25–4.66**	**0.01**
Road side	12	12	4.31	1.58–11.74	0.007
Don’t shop/Don’t remember	13	56	Ref	Ref	Ref
**Consumption of food/water prior to disease**					
Food sellers	14	78	**0.27**	**0.13–0.53**	** < 0.001**
Other household/Neighbors	25	58	0.64	0.35–1.17	0.19
Both	27	19	**2.10**	**1.04–4.23**	**0.05**
None	44	65	Ref	Ref	Ref
**Refuse disposal**					
Dumping in the backyard	49	105	2.10	0.81–5.42	0.18
Dumping in the bush	12	44	1.23	0.41–3.65	0.92
Combination of any of the above	43	43	**4.50**	**1.69–11.99**	**0.003**
Burning	6	27	Ref		

Factors associated with the cholera outbreak in Bauchi were: being a female (Odds ratio [OR] = 1.67, 95% CI = 1.05–2.66); occupation (Student) (OR = 2.96, 95% CI = 1.61–5.46); travel history (OR = 1.71, 95% CI = 1.03–2.81); contact with diarrhoea case (OR = 1.68, CI = 1.03–3.36); and attended a social gathering (OR = 0.10, CI = 0.05–0.23) see Table 3.

In the logistic regression model used, the following factors which were significant at bivariate analysis (*p* = 0.05) were subsequently added in the model, including contact with diarrheic patient, not attending social gathering, consuming food/water from food sellers, travel history, gender, waste disposal methods, occupation, source of drinking water and source of vegetables. After controlling for gender and using a stepwise elimination approach, only two factors remained statistically significant in the logistic regression model. In the final logistic regression model, attending social gathering or event (adjusted OR [aOR] = 2.04, 95% CI = 1.16–3.59) and drinking unsafe water (aOR = 1.74, 95%CI = 1.07–2.83) were risk factors for *Vibrio cholera* infection during the outbreak. Based on logistic regression analysis, we found that individuals who attended a social gathering or event (*p* = 0.02) and those with no access to safe drinking water (*p* = 0.03) were risk factors for the cholera outbreak (Table 4).

**Table 4:** Attending Social Gathering and Drinking of Unsafe Well Water as Risk Factors for Cholera Outbreak in Bauchi, September 2019

**Variables**	**Adjusted Odds Ratio**	**95% C.I**	***P*-Value**
**Age**			
> 5–20	1.53	0.81–2.89	0.19
21 and above			
**Contact with diarrheic patient**			
Yes	0.85	0.59–1.23	0.39
No			
**Attending social gathering or event**			
Yes	**2.04**	**1.16–3.59**	**0.01**
No			
**Sources of Food/water**			
Food sellers	0.91	0.70–1.18	0.48
Neighbors			
**History of travel anywhere in the 10 days**			
Yes	1.17	0.65–2.08	0.60
No			
**Gender**			
Female	1.29	0.80–2.10	0.30
Male			
**Waste disposal**			
Bush	1.05	0.82–1.34	0.70
Backyard			
**Occupation**			
Student	0.76	0.57–1.02	0.07
Civil servant			
**Type of drinking water**			
Well	**1.74**	**1.07–2.83**	**0.02**
Borehole-Tap water			
**Where do you shop for fruits and vegetables**			
Market	0.82	0.65–1.03	0.08
Roadside			
**Marital status**			
Single	0.96	0.50–1.85	0.90
Married			

### Environmental assessment

Based on our observations and findings in the community, the following were possible risk factors: Open defecation sites in about 66 communities within the metropolis; Poor environmental hygiene/sanitation as indiscriminate refuse dumpsites were seen all over the metropolis; limited access to safe water from the public water supply network; Poor personal hygiene practices; broken pipes and leakages especially along service lines; Open wells were the major sources of drinking water with most of them poorly protected; Building of latrines close to wells and exposed sewage as well as blocked drainages. We also inspected the homes of the cases for possible exposure factors.

## Discussion

Cholera is primarily a disease in underdeveloped and developing countries especially when there is a disruption in public sanitation services or normal balance of nature hence creating health problems as food and water supplies can become contaminated in such scenarios [12].

The index case was a young ‘Almajiri’ boy from an Islamic school. Most Islamic schools do not have toilet facilities for the pupils hence open defecation is the usual practice [13]. They lack access to potable water and mostly rely on open well water as their source of drinking water. The lack of these basic amenities coupled with exposure to poor sanitary and environmental conditions predisposes the group of children to infection through the feco-oral route [3, 13].

In Bauchi LGA being the epicenter of the outbreak, the case fatality rate of 0.27% was very low when compared with reports of similar outbreaks [1, 9]. This was probably due to the prompt setup of the cholera treatment center. However, the response of the RRT in curtailing and controlling the infection from spreading to other parts of the state was poor. Health education messages were also disseminated in affected communities via the mass media during the outbreak which led to better health-seeking behavior at treatment centers. Healthcare workers received refresher training on how to promptly identify cases; disinfect public and private toilets; chlorinate public and private wells and vaccination. Vaccination against cholera in Nigeria is usually not common [3]. Oral cholera vaccines were provided as a part of the outbreak response. For most individuals, this was the first time they had received the cholera vaccine with the support of NGOs. This is in agreement with reports of other studies where oral cholera vaccines were deployed during a cholera outbreak [14].

The outbreak affected different age groups in the affected areas, however, those who were > 5–20 years had the highest proportion of cases. This is consistent with findings of similar outbreaks in Nigeria where the most affected age group was 5–9 years [1, 3].

Gender was found to be associated with the outbreak as the odds of having cholera infection was 1.67 higher in females than in males and consistent with reports of other studies [9]. This was not surprising as women often make use of unhygienic water for cleaning, washing, cooking, or drinking when compared to men. The odds of infection amongst those that traveled 10 days before the infection was 1.71 higher than those that did not travel. This is similar to the report by Okeke et al. (2001) where population movement was shown to enhance the spread of the infectious agent to others and to different locations [15]. Both factors (gender and those that travelled 10 days before the infection) were not statistically significant by logistic regression.

By logistic regression analysis, the odds of infection amongst those that attended a gathering within the time of the outbreak were 1.93 higher than those that did not. Point source epidemics usually occur at gatherings such as a party or festivities. However, the subsequent spread of the infection may be suggestive of a common source epidemic since cholera can be spread feco-orally through ingestion of contaminated feed or water once the infection has been established. The index case was an ‘Almajiris’ boy from one of the Islamic schools in the State. These students usually live in poor sanitary and unhygienic environments making them prone to infections [16]. Attending social events was found to be a risk factor for acquiring cholera infection in the present study. This risk factor appears to be less informative. However, attending social events where food is served may be a good example of a point source epidemic.

Drinking unsafe well water was found to be a risk factor by bivariate analysis (OR = 3.52). The odds of infection in those who drank well water was 1.93 times higher than in those who drank borehole/tap water by multivariate analysis. This is consistent with findings of a similar study in Nigeria where drinking tap water was protective against the cholera outbreak and another study in Papua New Guinea that reported that piped water was protective against cholera [17, 18].

Those who dispose of their refuse in their backyard or the bush were found to be 4.50 times more likely to be infected with cholera when compared to those who burn their refuse. It is not uncommon to see refuse thrown in the backyard or in bushes being washed back by rainwater into rivers, surface wells, etc. thereby contaminating the water sources at various homes. However, this was not significant by logistic regression.

The prevention and control strategies practiced in Nigeria are multi-sectoral. Registration of cases, case management, and public health measures targeting personal hygiene and water treatment, as well as emergency responses as some of the Epidemic Preparedness and Response (EPR), measures put in place by both governmental and non-government agencies, have contributed to the reduction in case of fatality rates over the years and should be sustained [5]. However, to wholly curtail the infection, there is a need to explore more viable approaches. The endemicity of cholera in Nigeria provides a good avenue to use the surveillance system as an early alert for cholera outbreaks that will lead to a coordinated response. More importantly, it is necessary to introduce intervention measures that address the root problems of poor sanitation and unsafe water supplies in order to prevent future cholera epidemics [5, 19]. In this regard, perhaps, prevention of the disease is the best way to counter subsequent outbreaks. Measures such as boiling the water (for drinking and cooking purposes); sewages and drainage systems clearing; proper disposal of infected materials (such as waste products, clothing, and beddings); disinfection of infected facilities; treatment of infected fecal waste water produced by cholera victims and sterilization of utensils either by boiling or by using chlorine bleach will greatly reduce the incidence of cholera outbreaks. Studies have also indicated that the use of soap and handwashing promotion can achieve a 26 to 62% decrease in the incidence of diarrhea in developing countries [20–22]. Understanding the seasonality and location of outbreaks has been instrumental in providing appropriate guidance for cholera control activities in vulnerable areas [5, 19]. Health promotion activities including public enlightenment campaigns on cholera are important in ensuring that cholera outbreaks are controlled [5]. Health systems need to be strengthened with the provision of adequate manpower, equipment, drugs, and consumables [23]. To prevent future cholera outbreaks, there should be an improvement in surveillance systems, communication, transport, and mechanisms for quick intervention [24].

### Limitation of the study

Individuals used as controls were not tested for Cholera. It is possible that these individuals may be asymptomatic for cholera infection and be counted as a control even though they could be a case. Our inclusion criterium was to have participants that are above 5 years who we can engage in some level of conversation about their activities in the last 3–4 days prior to the onset of the infection. In Northern Nigeria, most women are indoors (due to religious beliefs) and so children can roam about with or without parental supervision. For this reason, we could not provide information for the age groups 0–5 years.

## Conclusions

The 2019 cholera outbreak in Bauchi State was investigated and the index case was found to be from an ‘almajiri’ student in Bauchi LGA. Thereafter, the infection was reported to have spread to other LGAs in the State. Attending social gatherings (aOR = 2.04) and drinking unsafe water (OR = 1.74) were found to be the risk factors for the cholera outbreak. The prompt setting up of the cholera treatment center in response to cases played a critical role in reducing the number of deaths recorded when compared with previous outbreaks. However, there was delayed intervention from the State's rapid response team. The Public health intervention instituted by the state includes chlorination of wells and distribution of water guard (1% chlorine solution) bottles to households and public education on cholera prevention.

We recommend based on our findings, organizing regular health talks on proper hand hygiene and chlorination of existing wells in the affected communities. The State government should improve the public water supply network for the inhabitants of the Bauchi metropolis and its environs, construct boreholes or protected wells as well as the establishment of good waste disposal systems. In addition, state government should provide Oral Cholera Vaccines as well as recruit more Environmental Health workers (Dubagari) to enforce hygiene and sanitation laws.

## Supplementary Information

**Additional file 1.**

## Data Availability

The datasets used and analyzed during the current study are available from the corresponding author on reasonable request. All data generated during this study are also included in this published article [and its supplementary information files].

## Abbreviations

ATBUTH: Abubakar Tafawa- Balewa University Teaching Hospital
AOR: Adjusted Odds Ratio
AR: Attack rate
BASPHCDA: Bauchi State Primary Health Care Development Agency
CFR: Case fatality rate
CI: Confidence interval
DSNO: Disease Surveillance and Notification Officer
LGA: Local Government Area
OR: Odds Ratio
RRT: Rapid Response Team
